# Effect of half adult dose of oral Rifampicin (300mg) in patients with idiopathic central serous chorioretinopathy

**DOI:** 10.12669/pjms.325.10755

**Published:** 2016

**Authors:** Muhammad Saim Khan, Murtaza Sameen, Arshad Ali Lodhi, Munawar Ahmed, Noman Ahmed, Mustafa Kamal, Sameen Afzal Junejo

**Affiliations:** 1 Muhammad Saim Khan, Department of Ophthalmology, Armed Forces Institute of Ophthalmology (AFIO), Rawalpindi, Pakistan; 2 Murtaza Sameen, Department of Ophthalmology, Armed Forces Institute of Ophthalmology (AFIO), Rawalpindi, Pakistan; 3 Arshad Ali Lodhi, Department of Ophthalmology, Liaquat University of Medical and Health Sciences, Jamshoro, Hyderabad, Pakistan; 4 Munawar Ahmed, Department of Ophthalmology, Liaquat University of Medical and Health Sciences, Jamshoro, Hyderabad, Pakistan; 5 Noman Ahmed, Department of Ophthalmology, Liaquat University of Medical and Health Sciences, Jamshoro, Hyderabad, Pakistan; 6 Mustafa Kamal, Department of Ophthalmology, Liaquat University of Medical and Health Sciences, Jamshoro, Hyderabad, Pakistan; 7 Sameen Afzal Junejo, Department of Ophthalmology, Liaquat University of Medical and Health Sciences, Jamshoro, Hyderabad, Pakistan

**Keywords:** Central serous chorioretinopathy, Macular thickness, OCT, Rifampicin

## Abstract

**Objectives:**

To evaluate the effect of half adult dose of oral Rifampicin on mean change in best corrected visual acuity and central macular thickness in patients with central serous chorioretinopathy.

**Methods:**

Thirty-eight eyes of 31 patients with idiopathic central serous chorioretinopathy (CSCR) were registered. Unaided Visual acuity, best corrected visual acuity was documented and detailed slit lamp examination along with dilated ophthalmoscopy was performed. All subjects were treated with oral Rifampicin 300 mg (half adult dose) daily for 03 months. Patients underwent a complete ocular and systemic examination as well as central macular thickness (CMT) measurement by optical coherence tomography (OCT) every month after starting treatment until four months. Fundus fluorescein angiography (FFA) was performed in recurrent cases. Liver function tests were carried out prior to the treatment and during follow up period.

**Results:**

A total of 38 eyes of 31 patients (24 males, 07 females) were included in the study. Mean age of patients was 36.16±3.19 years (range 30-44). Mean best corrected visual acuity (BCVA) before treatment was 0.56±0.11 and improved to 0.47±0.14 at 04 weeks (P<0.001) of treatment. The mean CMT at the time of presentation was 494.39±96.29 um and was decreased to 306.90±50.71 um after 04 weeks of treatment (P<0.001). The mean induced reduction in CMT was 187.48±122 um (P<0.001) while that in BCVA 0.41±0.16 at 04 weeks of treatment (P<0.001). Liver function tests were within normal range before and after the treatment.

**Conclusion:**

Half adult dose rifampicin (300mg) is effective and safe in treatment of central serous chorioretinopathy without causing any systemic imbalance.

## INTRODUCTION

Central serous retinopathy (CSR), also known as central serous chorioretinopathy (CSCR), a common retinal disorder which causes visual impairment was first introduced by Albrecht von Graefe in 1866 as ‘recurrent central retinitis’ (von Graefe, 1866) and later Donald Gass coined the term ‘central serous chorioretinopathy’ for its typical clinical presentation.^1^,^2^ It is characterized by the development of neurosensory retinal detachment from retinal pigment epithelium (RPE) mainly underneath macula with accumulation of sub retinal serous fluid secondary to a single, multifocal or diffuse area of leakage in RPE.^3^,^4^ Young healthy males with age ranging from 30-50 years are more commonly affected and male to female ratio varies from 2.32:1 to 6:1 in various global populations. The incidence of CSCR is higher in Asians than Caucasians and Hispanics, whereas African Americans are affected to a lesser extent.^5^-^6^ The disease is usually unilateral.

Patients of CSCR usually present with mild deterioration of central vision, metamorphosis, scotoma and/or induced hypermetropia secondary to central macular elevation.^7^ The exact mechanism involved in the etiology and evolution of CSCR remains unclear, however various risk factors have been associated with its development. Studies have shown the association of psychological stress, type A personality, endogenous/exogenous steroids, respiratory tract infection with elicobacter pylori, pregnancy and untreated hypertension with CSCR.^8^-^10^ Although diagnosis of CSCR is made by clinical examination, various investigation modalities such as Optical coherence tomography (OCT), and fundus fluorescein angiography (FFA) are used to confirm the diagnosis and monitor the disease. The advent of indocyaninegreen angiography (ICG) has revolutionized the understanding of CSCR pathogenesis and can be useful in making a treatment decision. It is by virtue of ICG that choroidal vascular system compromise and leakage has been considered as a fundamental event in the pathogenesis of this disorder, which was once considered as merely an RPE defect.^11^

Central serous chorioretinopathy resolves spontaneously within 3-6 months with good visual outcome and usually patients with first episode are observed. However 50% of the patients have recurrences and 15-20% progress to chronic form which has relatively poor visual outcome.^12^ Various treatment modalities such as standard laser photocoagulation, micro-pulse diode laser photocoagulation, trans-pupillary thermotherapy (TTT), photodynamic therapy (PDT)and multiple anti-corticosteroids agents have been proposed for the treatment of CSCR.^13^ A recent study documented mineralocorticoid receptors in choroidal tissue, and hypothesized that glucocorticoids activate mineralocorticoid receptors and may cause or aggravate CSCR manifestations [Zhao M et al 2012].^14^ As glucocorticoids are implicated in the development of CSCR, glucocorticoid inhibition with rifampicin has been suggested as a potential treatment modality.

Rifampicin, an antibacterial drug widely used in the treatment of tuberculosis and leprosy, is known to induce cytochrome P4503A4, therefore, having the potential to alter the metabolism of steroids [Guengerich FP, 1999], and may lead to an improvement in CSCR manifestations. Rifampicin can effectively overcome CSCR by inducing catabolism of endogenous steroids, however the normal dose 600mg daily has been associated with complications such as hepatotoxicity.^13^

The rationale of conducting this study was the possible risk of complications associated with normal full dose rifampicin. Rifampicin 300 mg twice daily has been used in a small series to treat chronic CSCR with success.^15^

The aim of this study was to evaluate the effect of half daily dose of oral rifampicin (300mg) treatment for a period of three months in patients with idiopathic CSCR with visual impairment. The primary outcome measures included change in BCVA and change in the choroidal thickness after treatment.

## METHODS

This clinical observational study was carried out at the department of Ophthalmology Liaquat University of Medical and Health Sciences (LUMHS), Hyderabad, Pakistan between Jan 2013 and June 2015. A total of 38 eyes of 31 patients with age ranging from 30 to 44 years, and diagnosed as having idiopathic CSCR were included in the study by non-probability (purposive) sampling. Idiopathic CSCR was defined as a localized neurosensory retinal detachment associated with a focal leak or leaks at the level of the retinal pigment epithelium by fluorescein angiography. Patients with evidence of serous elevations secondary to other ocular conditions such as optic disc pit, multifocal choroiditis, posterior scleritis, choroidal mass or associated systemic illnesses were excluded from the study.

The study was approved by the local ethical committee and carried out in accordance with the Declaration of Helsinki. Informed verbal/written consent was taken from all registered subjects. Demographic details and blood group were noted. Unaided distant visual acuity (UDVA), visual acuity by using plus one convex spherical lens and best-corrected visual acuity (BCVA) was documented followed by detailed slit lamp examination after pharmacological dilation. Optical coherence tomography (OCT) an imaging technique was performed to confirm the diagnosis and note the central macular thickness (CMT). Fundus Fluorescein Angiography was done in recurrent cases. The registered subjects were started with an oral 300 mg daily dose of Rifampicin for 3 months. Patients were reviewed at 4 weeks, 8 weeks, and 4 months for detailed ocular and systemic examination as well as measurement of central macular thickness by OCT. Liver function tests such as Alanine aminotransferase (ALT), Aspartate aminotransferase (AST) and Alkaline phosphatase (ALP) were carried out before starting the treatment and every four weeks treatment follow up.

### Laboratory tests

Systemic laboratory work-up included a complete blood count and liver function tests at baseline and at 1, 2, and 3 months post treatment initiation. Following the confirmation of normal liver function tests at baseline, the patients were included in the study and received oral Rifampicin at a dosage of 300 mg daily for 3 months.

### Statistical analysis

We carried out the statistical analysis using statistical package for social sciences (SPSS 21.0) for windows. Continuous data such as Age, BCVA, CMT were described in terms of mean ± SD (standard deviation). The induced change in BCVA and CMT after comparing the pre-treatment and 04 weeks, 08 weeks and 04 months after starting the treatment were evaluated statistically with paired sample test (p < 0.05 significance level). Frequency distribution of categorical variables was also analyzed statistically (*P<*0.05 significance level).

### Ethics Approval

Ethics and research committee of Liaquat University of Medical and Health Sciences/Jamshoro, Hyderabad, Pakistan approved the study.

## RESULTS

A total of forty-one (41) subjects (30 [73.1%] male, 11[26.9%] female) were enrolled. Four (9.75%) were lost to follow up. Six (14.63%) patients showed significant abnormal liver function tests and thus were referred to medical consultant for further investigation and treatment.

Thirty- eight eyes of 31 patients (24 males, 7 females) were registered. Of these, seven patients had bilateral involvement, while the rest were unilateral cases, 14 were right and 10 were left eyes. Ten patients belonged to rural background while 21were from urban areas. Table-I shows the frequency distribution of various categorical variables. Liver function tests of all the registered subjects were within normal range before starting the treatment (Mean ALT= 32 ^±^ 2 IU/L, AST= 35 ^±^ 2 IU/L, ALP = 86 ^±^ 12 IU/L) and remained so after treatment at 4 weeks (Mean ALT= 30^±^ 3 IU/L, AST= 31^±^ 4 IU/L, ALP = 90^±^6 IU/L), 8 weeks (Mean ALT= 34^±^ 3 IU/L, AST= 34^±^ 3 IU/L, ALP = 84^±^10 IU/L) and 4 months (Mean ALT= 30^±^ 3 IU/L, AST= 36^±^2 IU/L, ALP = 81^±^11 IU/L).

**Table-I:** Frequency distribution of various categorical variables: n=31.

*Variables*		*Frequency*	*Percentage (%)*
Gender	Males	24	77.4
	Females	07	22.6
Laterality	Right	14	45.2
	Left	10	32.3
	Bilateral	07	22.6
Primary/Recurrent	Primary	23	74.2
	Recurrent	07	22.6
Blood Groups	O+ve	08	25.8
	B+ve	20	64.5
	A+ve	03	9.7

Blood grouping assessment revealed B^+v^ in 64.5% subjects, O^+ve^ in 25.8% and remaining 9.7% were A^+ve^. Mean age of patients was 36.16 ^±^ 3.19 years with a range from 31 to 42 years.

**Fig.1:**
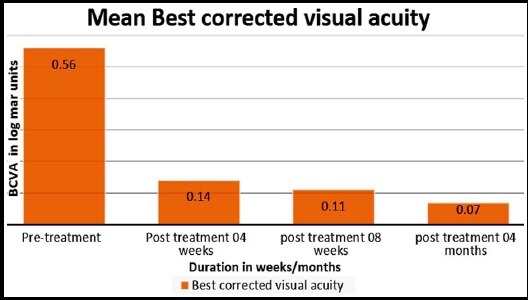
Best corrected Visual acuity (BCVA) before and after treatment at four months.

**Fig.2:**
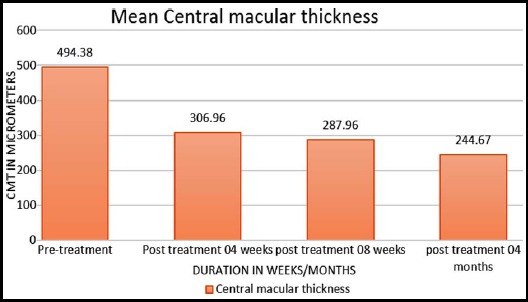
Central Macular thickness (CMT) before and after treatment at four months.

Mean best corrected visual acuity (BCVA) was 0.56± 0.116 with a range of 0.00 to 0.77 before starting the treatment while after treatment at 04 weeks visual acuity was of 0.147± 0.148. The mean CMT at the time of presentation was 494.39±96.2 um while it was 306.90 after 04 weeks of starting half dose (300 mg daily) Rifampicin. The mean induced reduction in CMT was 187.48±122 um while that in BCVA was 0.41± 0.16 (Table-II). The mean change in best corrected visual acuity and central macular thickness over a period of 04 months of treatment is shown in Fig.1 and Fig.2 respectively.

**Table-II:** Mean, standard deviation and induced change of BCVA and CMT before and after treatment at 04 weeks.

	*Mean ± SD*	*P - value*
Pre-treatment BCVA	0.56 ± 0.11	P < 0.001
Pre-treatment CMT	494.39 ± 96.29	P < 0.001
Post-treatment BCVA	0.47 ± 0.14	P < 0.001
Post-treatment CMT	306.90 ± 50.71	P < 0.001
Induced Change in BCVA at 04 weeks	0.41 ± 0.16	P = 0.001
Induced change in CMT at 04 weeks	187.48 ± 122.01	P = 0.001

BCVA = Best Corrected Visual Acuity, CMT = Central Macular Thickness.

## DISCUSSION

Rifampicin is an antibiotic that has been widely used as first line drug in treatment of tuberculosis along with Isoniazid, Pyrazinamide and Ethambutol.^14^ The drug has also been known to have anti-oxidative, and anti-apoptotic properties and to induce cytochrome P-450 enzyme in liver. The role of Rifampicin in CSCR was introduced by Ravage and Packo when they found resolution of CSCR in a patient with tuberculosis.^15^-^17^ This was followed by first ever case report published by Seinle NC and his colleagues who also proposed the metabolism of endogenous steroids by Rifampicin induced cytochrome P450 as basic mechanism involved in improvement of CSCR patients.^15^

Shulman et al, in their study of 12 patients with chronic CSCR revealed significant improvement in vision as well as resolution of subretinal fluid at one month and this improvement was maintained over a period of 3-6 months. However, 16.6 % of patients developed adverse effects secondary to deranged liver functions and treatment was prematurely stopped in these patients.^18^ Drug induced liver injury (DILI) has been regarded as a potential side effect of Rifampicin. The mechanism involved is oxidative stress induced by Rifampicin that leads to hepatocyte injury and raised liver enzymes in serum.^19^,^20^

We, like Shulman et al, found significant improvement in vision as well as central retinal thickness at 4 weeks after starting the treatment with half daily dose of Rifampicin. The improvement in vision as well as CMT was seen in 89.4% (34 out of 38 eyes) in our study, which was much higher than what Shulman et al has concluded.^18^ This difference can be due to different patient selection; most of our patients (74.2%) were recent onset cases of CSCR, while Shulman and his colleagues included all cases of chronic CSCR in their study. We did not find statistically significant improvement in 10.5% (4 out of 38 eyes) of our patients. In this study, we also found high prevalence of B+ve blood group in our patients (Table-III).

**Table-III:** Multivariate analysis of various risk factors.

*Variables*	*Frequency (n=31)*	*Percentage*
Systemic steroids intake	0	0.00 %
Sytsemic antibiotics intake	01	3.22 %
Pregnancy	0	0.00
Systemic hypertension	01	3.22%
Diabetes	01	3.22%
Type A personality	0	0%
Psycological stress	0	0%

Hepatotoxicity, which is a known adverse effect of systemic Rifampicin therapy at a dose of 600 mg per day,^19^,^20^ was not seen in any of our patients having daily dose of 300mg. Liver function tests which were performed before and during the follow up period remained within normal range.

**Fig.3:**
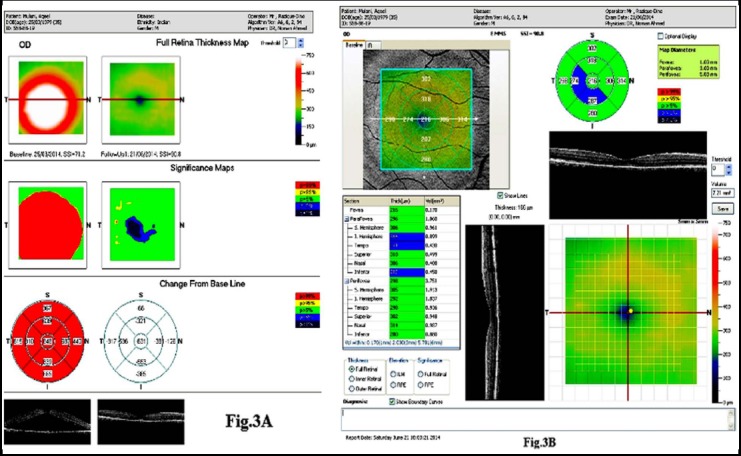
A and B: OCT and Fundus photograph of a patient before treatment (CMT=848 microns) (Fig:3A) and 3 months of commencement of treatment (CMT= 216 microns) (Fig:3B) with oral rifampcin 300 mg half adult dose daily.

In our study, we used half daily dose of rifampicin, which has not been reported in the literature so far except in a single case report. The results in new as well as recurrent cases of CSCR at the end of 3 months were statistically and clinically significant (Fig.3 A and B). There was marked improvement in the visual acuity and complete resolution of subretinal fluid was seen in around 90% of the patient as evidenced by optical coherence tomography. Moreover, we did not observe any side effects or complications with this treatment. Our study showed significant positive results and our findings are important and worth reporting. Whether to use half daily dose of Rifampicin in all cases of central serous retinopathy or recurrent/chronic cases is an area that needs to be further evaluated. Furthermore, studies on a larger cohort and for a longer period of time, considering all the risk factors and confounding variables should be carried out to generate more comprehensive results. In conclusion, half daily dose of Rifampicin is an effective and safe treatment in patients with central serous chorioretinopathy without affecting the normal hepatic function.

### Limitations of the study

This includes the fact that new cases of CSCR were major proportion of our sample, making around 74% in comparison to recurrent cases that were only 26%. Therefore, we could not consider the natural time for disease resolution that is supposed to occur in more than 50% of the cases. Secondly we could not rule out the confounding role of stress therapy, lifestyle changes and anxiety related management that patients may also be undergoing. Thirdly we could only follow the patients for a relatively shorter period of four months after the treatment.
